# Two-Component Signal Transduction System SaeRS Positively Regulates *Staphylococcus epidermidis* Glucose Metabolism

**DOI:** 10.1155/2014/908121

**Published:** 2014-01-23

**Authors:** Qiang Lou, Yijun Qi, Yuanfang Ma, Di Qu

**Affiliations:** ^1^Laboratory of Cellular and Molecular Immunology, Henan University, Kaifeng 475004, China; ^2^Key laboratory of Medical Molecular Virology of Ministry of Education and Ministry of Public Health, Institute of Medical Microbiology and Institutes of Biomedical Sciences, Shanghai Medical College of Fudan University, 138 Yixueyuan Road, Shanghai, 200032, China

## Abstract

*Staphylococcus epidermidis*, which is a causative pathogen of nosocomial infection, expresses its virulent traits such as biofilm and autolysis regulated by two-component signal transduction system SaeRS. In this study, we performed a proteomic analysis of differences in expression between the *S. epidermidis* 1457 wild-type and *saeRS* mutant to identify candidates regulated by saeRS using two-dimensional gel electrophoresis (2-DE) combined with matrix-assisted laser desorption/lonization mass spectrometry (MALDI-TOF-MS). Of 55 identified proteins that significantly differed in expression between the two strains, 15 were upregulated and 40 were downregulated. The downregulated proteins included enzymes related to glycolysis and TCA cycle, suggesting that glucose is not properly utilized in *S. epidermidis* when *saeRS* was deleted. The study will be helpful for treatment of *S. epidermidis* infection from the viewpoint of metabolic modulation dependent on two-component signal transduction system SaeRS.

## 1. Background

With all kinds of artificial medical materials commonly used, the opportunistic pathogen *Staphylococcus epidermidis* has become a major pathogen of nosocomial infection [1, 2]. *S. epidermidis* pathogenesis is associated with its ability to form biofilms on the surface of medical indwelling materials [3]. The biofilms have the ability of resistance to antibiotics and the host immune system.

Biofilm formation proceeds in two distinct developmental phases: primary attachment of staphylococcal cells to a polystyrene surface followed by bacterial accumulation in multiple layers. During the initial stage of biofilm formation, extracellular DNA (eDNA) is critical for bacterial attachment [4]. Extracellular DNA release from *S. epidermidis* is related to AtlE-mediated bacterial autolysis. During the bacterial accumulation phase in *S. epidermidis*, biofilm formation is mediated by polysaccharide intercellular adhesin (PIA) synthesized by *icaADBC* operon-encoded enzymes [5].

Glucose can induce the* S. aureus* biofilm formation by inducing *ica* gene. The addition of glucose to growth media triggered biofilm formation in *S. aureus* strain SA113 [6], increased the expression of genes and enzymes of the glycolytic pathway, while genes and proteins of the tricarboxylic acid (TCA) cycle, required for the complete oxidation of glucose, were repressed via catabolite control protein A (CcpA) [7]. Deletion of CcpA, which regulates gene expression in response to the carbon source, abolished the capacity of SA113 to form a biofilm. Biofilm formation is closely related to bacteria energy metabolism and cell wall synthesis [5]. Disintegration of *S. epidermidis *biofilms under glucose-limiting conditions depends on the activity of the alternative sigma factor sigmaB [8].

Glycolysis that is the principal pathway of glucose metabolism occurs in the bacterial cytoplasm where glucose is oxidized to pyruvate (in aerobic condition) or lactate (in anaerobic condition) and generates energy in the form of ATP. The accumulation of organic acids in the culture medium occurred coincident with a decreased pH. The pH change coincided with changes in cell viability, cell ATP, and the ability to produce acidity by glucose fermentation [9]. Glyceraldehyde 3-phosphate dehydrogenase is an essential enzyme that catalyzes the sixth step of glycolysis and thus serves to break down glucose for energy and carbon molecules [10]. Glucose metabolism of* Escherichia coli* strain NP315 *fda* (encoding fructose-1, 6-diphosphate aldolase) and *gnd* (encoding 6-phosphogluconate dehydrogenase) mutant was decreased [11].

Staphylococcal biofilm formation is a complicated process that is regulated by two-component signal transduction systems (TCSs) [12, 13]. Prokaryotic TCSs modulate gene expression in response to different environmental signals [14]. SaeRS TCS was originally identified in *Staphylococcus aureus* as a transposon mutant system deficient in the synthesis of several exoproteins, including *α*- and *β*-hemolysin and coagulase [15]. In *S. aureus* COL, proteomic approach showed that extracellular proteins were influenced by a mutation in *saeS* [16]. In a previous study of the *S. epidermidis* SaeRS TCS, a *saeR* deletion mutant exhibited a lower anaerobic growth rate, a significantly reduced rate of nitrate utilization, and a slightly higher biofilm-forming ability compared to the parental strain [17]. In *S. epidermidis*, the TCS SaeRS is known to be involved in biofilm formation. Furthermore, SaeRS was found to be involved in *S. epidermidis* autolysis, resulting from altered bacterial eDNA release [12].

In the present study, the influence of the TCS SaeRS on global gene expression in *S. epidermidis* was investigated by proteomic approach. SaeRS positively regulates the proteins expression involved in glucose metabolism. Furthermore, we show that glucose metabolism by decreasing the pH value affects biofilm-forming ability and bacterial cell viability.

## 2. Materials and Methods

### 2.1. Bacterial Strains, Plasmids, and Media

The bacterial strains used in this study are *S. epidermidis* wild-type (WT),* S. epidermidis saeRS* mutant (SAE), and *S. epidermidis saeRS* complemental strain (SAEC). *S. epidermidis* cells were grown at 37°C in BM medium (Tryptone 10 g, Yeast extract 5 g, NaCl 5 g, K_2_HPO_4_ 1 g, and Glucose 1 g/L medium) or tryptic soy broth (TSB) (Oxiod, Basingstoke, Hampshire, England) supplemented with antibiotics when necessary. Spectinomycin (spc) was used at 300 *μ*g/mL for *S. epidermidis*.

### 2.2. Sample Preparation for Two-Dimensional Gel Electrophoresis (2-DE)

SAE and WT were grown until the exponential phase (OD600 = 1.6) in TSB medium and the bacteria (7.56 × 10^9^ cells of each) were harvested by centrifugation at 7,000 g for 10 min at 4°C. The bacterial pellets were solubilized with lysis buffer (7 M urea, 2 M thiourea, 2% (w/v) CHAPS, and 50 mM dithiothreitol (DTT), 2% (v/v) pH 4–7 immobilized pH gradient (IPG) buffer (Amersham Biosciences) containing 1% protease inhibitor cocktail (Roche) and 1 mM PMSF (Sigma)) and then sonicated on ice for 12 cycles, each consisting of 5 s pulse and 10 s pause. After centrifugation at 7,000 g for 1 h at 4°C, the supernatants of lysates were divided into aliquots and the protein concentrations were determined by the Bradford assay. Then, aliquots were stored at −80°C for further analysis.

### 2.3. Two-Dimensional Gel Electrophoresis and Image Analysis

The 2-DE gels were performed using 24 cm IPG strips (pH 4–7, GE Healthcare) in Ettan IPGphor isoelectric focusing system (Amersham Biosciences) plus Ettan-Dalt Six system (Amersham Biosciences) according to the manufacturer's instructions. To compensate the variability of gel electrophoresis, at least three replicate gels were performed for each group. In the first dimensional isoelectric focusing (IEF), 120 *μ*g proteins of each sample were diluted to 450 *μ*L with rehydration buffer containing 8 M urea, 2% (w/v) CHAPS, 50 mM DTT, and 0.5% (v/v) ampholyte (pH 4–7, Amersham Biosciences), and IPG strips were allowed to rehydrate in the above solution under mineral oil. IEF was performed as follows: 30 V for 6 h (active rehydration), 60 V for 6 h (active rehydration), 500 V for 2 h, rapid, 1,000 V for 2 h, rapid, 4,000 V for 2 h, linear, linear ramping to 8,000 V for 2 h, and finally 8,000 V for about 7 h with a total of 64 KVh at 20°C. Then the IPG strips were incubated in equilibration buffer (75 mM Tris-HCl (pH 8.8), 6 M urea, 29.3% (v/v) glycerol, 2% (w/v) SDS, and 0.002% (w/v) bromophenol blue) containing 2% (w/v) DTT for 15 min with gentle agitation, followed by incubation in the same equilibration buffer supplemented with 2.5% (w/v) iodoacetamide for 15 min at room temperature. The second dimension SDS-PAGE was performed on 1 mm thick 12.5% polyacrylamide vertical gels in Ettan-Dalt Six system using 5 W/gel for 30 min and followed by 12 W/gel at 10°C until the bromophenol blue dye front reached the end of the gels. The gels were stained by a modified silver staining method compatible with MS analysis and scanned at 300 dpi (dots/inch) using ImageScanner (UMAX, Amersham Biosciences). Images were captured and analyzed by ImageMaster 2D platinum 6.0 software (Amersham Biosciences). The percentage of the volume of the spots representing a certain protein was determined in comparison with the total proteins present in the 2-DE gel. To select differentially expressed protein spots, quantitative analysis was performed using the Student's *t*-test to compare the percentage volumes of spots between SAE and WT groups. The differentially expressed protein spots with *P* values less than 0.05 were considered significant differences, and at least 1.5-fold difference in percentage of the volume for each spot was set as a threshold. These protein spots were selected and subjected to in-gel tryptic digestion and identification by MS.

### 2.4. In-Gel Tryptic Digestion

The differentially expressed protein spots were manually excised from the sliver-stained gels (each gel of 120 *μ*g protein) and placed into a 96-well microplate. The gel pieces were destained with a solution of 15 mM potassium ferricyanide and 50 mM sodium thiosulfate (1 : 1) at room temperature for 10 min, then washed twice with deionized water, each for 30 min, and dehydrated in 80 *μ*L of acetonitrile (ACN) twice. Then the samples were swollen in a digestion buffer containing 25 mM NH_4_HCO_3_ and 12.5 ng/*μ*L trypsin (Promega) at 4°C after 30 min incubation and incubated at 37°C for more than 12 h. The peptide mixtures from the gel were extracted twice using 0.1% trifluoroacetic/50% ACN at room temperature, resuspended with 0.7 *μ*L matrix solution (*α*-cyano-4-hydroxycinnamic acid (Sigma) in 0.1% trifluoroacetic, 50% ACN), and allowed to dry in air under the protection of N_2_.

#### 2.4.1. Mass Spectrometric Analysis and Database Searching

The peptide mixtures from samples were analyzed by 4700 MALDI-TOF/TOF Proteomics Analyzer (Applied Biosystems). The UV laser was operated at a 200 Hz repetition rate with wavelength of 355 nm. The accelerated voltage was operated at 20 kV. Myoglobin digested by trypsin was used to calibrate the mass instrument with internal calibration mode. All acquired spectra of samples were processed using 4700 series Explore software (Applied Biosystems) in a default mode. The parent mass peaks with mass range 700–3200 Da and minimum S/N 20 were picked out for tandem TOF/TOF analysis. Combined MS and MS/MS spectra were submitted to MASCOT (V2.1, Matrix Science) by GPS Explorer software (V3.6, Applied Biosystems) and searched with the following parameters: NCBInr database (release date: November 2009), taxonomy of bony vertebrates or viruses, trypsin digest with one missing cleavage, none fixed modifications, MS tolerance of 100 ppm, MS/MS tolerance of 0.6 Da, and possible oxidation of methionine. Known contaminant ions (human keratin, tryptic autodigest peptides, etc.) were excluded. MASCOT protein scores (based on MS and MS/MS spectra) with greater than 72 were considered statistically significant (*P* < 0.05). The individual MS/MS spectrum with statistically significant (confidence interval >95%) and best ion score (based on MS/MS spectra) was accepted.

### 2.5. Quantitative Real-Time PCR Analysis

Total RNA was extracted from the SAE and WT after incubation for 12 h. For each RNA sample, duplicate reverse transcription reactions were performed using reverse transcriptase MMLV (Promega, Madison, USA), with a control without reverse transcriptase. RT real-time sequence-specific detection was performed using SYBR *Premix Ex Taq* (TaKaRa) and relative quantification of gene expression was calculated using the comparative cycle threshold method as described for the ABI 7500 real-time PCR system (Applied Biosystems, California, USA) by the manufacturer's instructions. The housekeeping gene* gyrB* was used as an endogenous control. Gene-specific primers were designed according to the gene sequences at GenBank (Accession no. CP000029) (Table 1). All samples were analyzed in triplicate and normalized against *gyrB* gene expression.

### 2.6. Biofilm and PIA Assay

The biofilm-forming ability of *S. epidermidis* strains was determined by the microtiter-plate test as described by Christensen. Briefly, overnight cultures of *S. epidermidis *were diluted 1 : 200 in BM medium and inoculated into wells of polystyrene microtiter plates (200 *μ*L per well) at 37°C for 24 h. Glucose (G8270, Sigma) was added into BM medium at 0.125%, 0.25%, 0.5%, and 1% (g/mL), respectively. After incubation, the wells were gently washed three times with 200 *μ*L PBS and stained with 2% crystal violet for 5 min. Absorbance was determined at 570 nm.

*S. epidermidis* cells for PIA immunoblot assays were grown in 6-well plates (Nunc, DK-4000 Roskitde, Denmark) under static conditions at 37°C for 24 h. The cells were scraped off and resuspended in 0.5 M EDTA (pH 8.0). The supernatant was treated with proteinase K (final concentration 4 mg/mL; Roche, MERCK, Darmstadt, Germany) for 3 h (37°C). Serial dilutions of the PIA extract were then transferred to a nitrocellulose membrane (Millipore, Billerica, MA) using a 96-well dot blot vacuum manifold (Gibco). The air-dried membrane was blocked with 3% (wt/vol) bovine serum albumin and subsequently incubated with 3.2 *μ*g/mL wheat germ agglutinin coupled to horseradish peroxidase (WGA-HRP conjugate; Lectinotest Laboratory, Lviv, Ukraine) for 1 h. Horseradish peroxidase (HRP) activity was visualized via chromogenic detection. The gray scale of the spots corresponding to PIA was quantified using the Quantity One software.

### 2.7. CFU Count and Acidity Determination

Overnight cultures of *S. epidermidis *were diluted 1 : 200 in BM medium with or without 0.125%, 0.25%, 0.5%, and 1% (g/mL) glucose and incubated at 37°C for 24 h. Colony forming units (CFU) on TSA plates were further counted with serial dilutions of each sample plated on 6 agar plates. Then, the cultures were centrifuged. The pH of the supernatant was determined by using a pH meter (FE20, Mettler-Toledo, Shanghai, China). Then, five drops of methyl red were added to the supernatant and then gently rolled to disperse the methyl red. The supernatant will turn yellow at neutral pH but turn red at pH < 4.0.

### 2.8. Statistical Analysis

Experimental data were analyzed with the SPSS software and compared using the Student's *t*-test. Differences with a *P* value of <0.05 were considered statistically significant.

## 3. Results

### 3.1. Protein Expression Profiles of *S. epidermidis* Affected by Two-Component Signal Transduction System SaeRS

**Figure 1:**
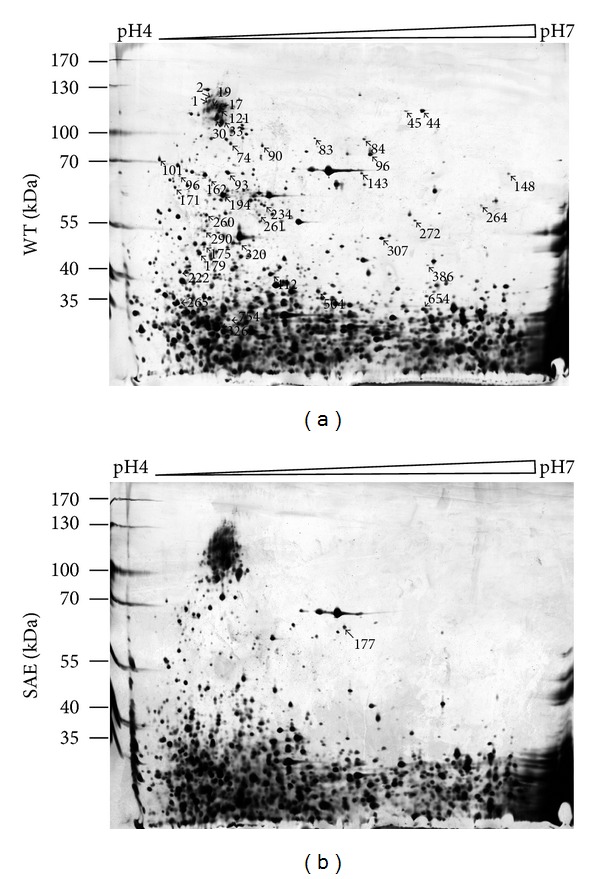
Identified spots on the 2D proteome map of SAE and WT. SAE and WT were grown in TSB medium at 37°C until the postexponential growth phase; the bacteria were then separated by centrifugation. Bacteria cell pellets were dissolved in lysis buffer and sonicated on ice. The 2-DE gels were performed using 24 cm immobilized dry strips (IPG, nonlinear, pH 4–7, GE Healthcare) and analyzed by ImageMaster 2D platinum 6.0 software (Amersham Biosciences). Differentially expressed protein spots on 2-DE gels were identified by MS/MS analysis and shown in gels with unique protein spot numbers. Protein spots were identified using a 4700 MALDI-TOF/TOF Proteomics Analyzer (Applied Biosystems, California, USA).

Two-dimensional gel electrophoresis (2-DE) was performed to analyze the expression of *S. epidermidis *proteins regulated by two-component signal transduction system SaeRS. The gel images were compared and analyzed. The patterns of protein expression in SAE and WT appeared largely similar but some clear differences in protein expression levels of certain protein spots were evident (Figure 1). Fifty-five protein spots were excised for further analysis by the LC-MS/MS. Of these, 15 spots were up-regulated, and 40 were downregulated (Table 2).

The 55 identified proteins that varied in expression levels showed diverse annotated functions (the identified proteins were functionally classified into the category of metabolic enzyme, biofilm formation, transcriptional regulator, chaperone, energy metabolism, or unknown function), including the enzymes involved in glycolysis (fructose-bisphosphate aldolase, glyceraldehyde-3-phosphate dehydrogenase, enolase and pyruvate kinase) and TCA cycle (succinyl-CoA synthetase alpha and beta subunits, and isocitrate dehydrogenase), biofilm forming-related proteins (accumulation-associated protein), stress response protein (heat shock protein 60 and dnaK protein), serine proteinase inhibitor (alpha-2-macroglobulin), cytoskeletal proteins (actin, tubulin, and twinstar), enzymes involved in energy metabolism (ATP synthase subunit B) and proteins involved in glucose transport (PTS system glucose-specific enzyme II A), and regulator of carbon metabolism (catabolite control protein A).

### 3.2. Enzymes

**Figure 2:**
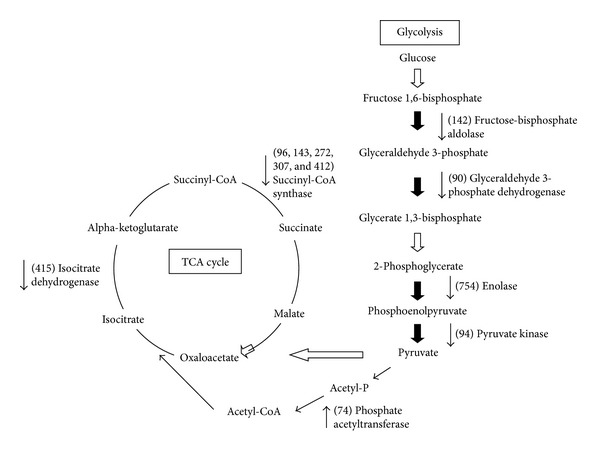
Carbohydrate metabolic pathways in the *saeRS* mutant under aerobic conditions. The following enzymes (identified in the 2-DE experiment) were found at lower levels in the *saeRS* mutant: fructose bisphosphate aldolase, glyceraldehyde 3-phosphate dehydrogenase, enolase, pyruvate kinase, succinyl-CoA synthase, and isocitrate dehydrogenase. Phosphate acetyltransferase was found at higher level in the *saeRS* mutant.

Approximately 40% of the identified proteins were defined as enzymes. Most of the identified enzymes were upregulated at the postexponential phase and categorized as involved in glucose metabolism.  Figure 2 showed that enzymes related to glycolysis (fructose-bisphosphate aldolase (GI: 27469074), glyceraldehyde 3-phosphate dehydrogenase (GI: 27467475), enolase (GI: 27467479), pyruvate kinase (GI: 27468291)), the TCA cycle (succinyl-CoA synthase (GI: 27467842), and isocitrate dehydrogenase (GI: 27468288)) were downregulated at the post-exponential phase. This is the first report that the expression of enzymes related to carbohydrate metabolism in *Staphylococcus *species is down-regulated by deletion of TCS SaeRS.

### 3.3. Real-Time RT-PCR of the Differentially Expressed Genes

**Figure 3:**
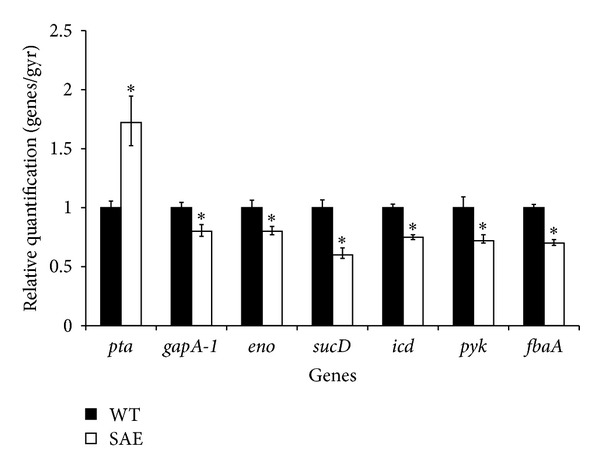
Quantitative transcript analysis of glucose metabolism-related gene transcript levels. Total RNA was extracted from SAE and WT grown in TSB medium at 37°C until the postexponential growth phase and the cDNA were then synthesized. The mRNA expression levels of glyceraldehyde 3-phosphate dehydrogenase (gapA-1), enolase (eno), succinyl-CoA synthase alpha subunit (sucD), isocitrate dehydrogenase (icd), pyruvate kinase (pyk), fructose-bisphosphate aldolase (fbaA), and phosphate acetyltransferase (pta) were determined using the beta-actin gene as an internal control. Data are shown as the mean ± S.D. Means with different letters are significantly different (*P* < 0.05).

To verify the differentially expressed proteins regulated by SaeRS at the transcriptional level, glucose metabolism-related genes were chosen for the quantitative real-time RT-PCR analysis. The *gyrB* gene was used as an internal control. The transcription of glyceraldehyde 3-phosphate dehydrogenase, enolase, succinyl-CoA synthase alpha subunit, isocitrate dehydrogenase, pyruvate kinase, and fructose-bisphosphate aldolase was significantly decreased in SAE, approximately 0.8-, 0.8-, 0.6-, 0.75-, 0.72-, and 0.7-fold lower than that in WT, respectively (Figure 3). The phosphate acetyltransferase transcription expression levels were significantly upregulated 1.72-fold over than those of the control.

### 3.4. Effect of *saeRS* Deletion on *S. epidermidis *Biofilm Formation in Response to Glucose

**Figure 4:**
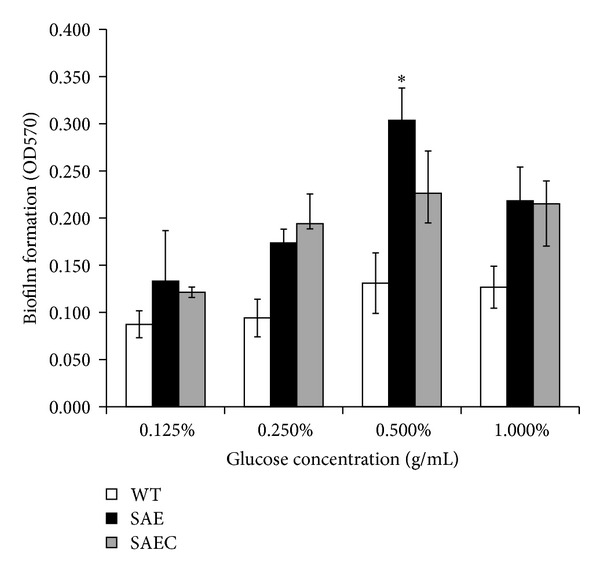
Effect of glucose on SAE, WT, and SAEC biofilm formation. SAE, WT, and SAEC biofilms were formed in the absence (black bars) or presence of glucose (0.125%, 0.25%, 0.5%, and 1%); then the biofilms were washed and then stained with crystal violet. Their retained biomass was quantified by measuring the absorbance of each well at 570 nm. Mean values and standard deviations from three independent experiments are shown, *P < 0.05.

As TCS SaeRS regulated glucose metabolism and biofilm formation-related genes expression, the effect of *saeRS* deletion on *S. epidermidis *biofilm formation in response to various concentrations of glucose was further examined. Upon the addition of glucose to the BM medium, the biofilm forming ability of SAE was significantly increased compared to the parental strain and reached the peak level at 0.5% (g/mL) glucose (Student's *t*-test, *P* < 0.05) (Figure 4). However, the biofilm forming ability of WT showed no obvious difference upon the stimulation of glucose. Although it did not reach the level of the wild-type strain, complementation of *saeRS* resulted in decreased biofilm formation.

### 3.5. Effect of *saeRS* Deletion on *S. epidermidis *Viability in Response to Glucose

**Figure 5:**
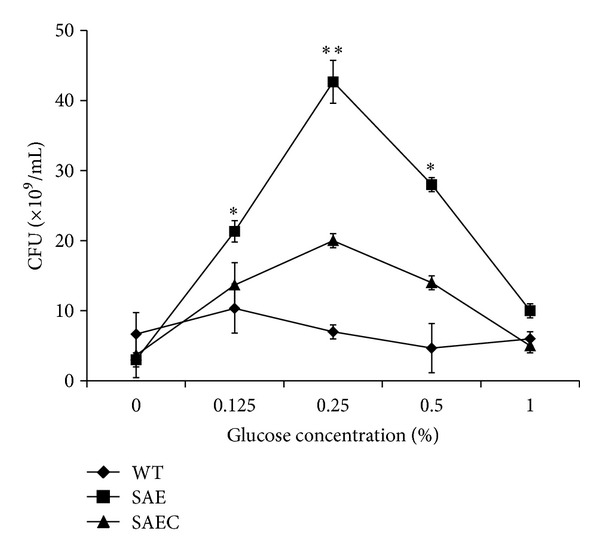
Viability of *S. epidermidis* 1457 in BM medium containing various concentrations of glucose. Overnight cultures of *S. epidermidis *were diluted 1 : 200 in BM medium with or without 0.125%, 0.25%, 0.5%, and 1% (g/mL) glucose and incubated at 37°C for 24 h. Colony forming units (CFU) on TSA plates were further counted with serial dilutions of each sample plated on 6 agar plates.

In our previous study, *saeRS* deletion in *S. epidermidis* decreased the bacterial cell viability. To investigate whether the decreased cell viability that resulted from *saeRS* deletion was affected by glucose metabolism, colony-forming unit (CFU) counts of SAE, WT, and SAEC in BM medium with or without glucose (0.125%, 0.25%, 0.5%, and 1%) were determined. Cultures were inoculated with approximately 10^4^ CFU/mL of each strain and incubated for 24 h. When cultured in medium containing 0.25% glucose, SAE cultures had the highest CFU count (4.23 × 10^10^ CFU/mL) compared to WT (7 × 10^9^ CFU/mL) and SAEC (2 × 10^10^ CFU/mL) (Figure 5); SAE showed the highest viability when grown in medium containing 0.25% glucose, compared with the other growth conditions.

### 3.6. Effect of *saeRS* Deletion on Acidity in Response to Glucose

**Figure 6:**
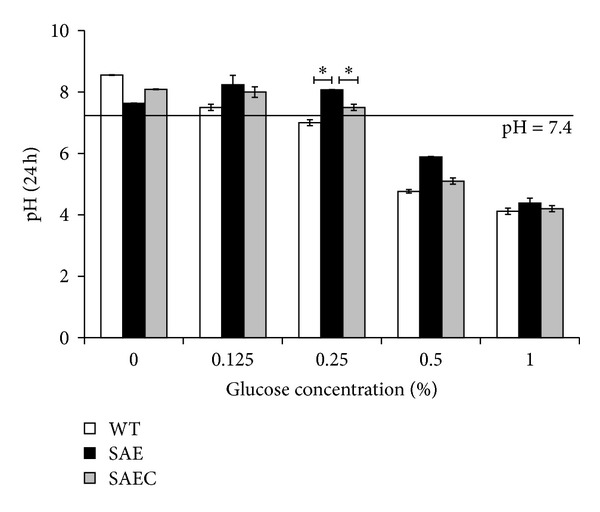
pH value of *S. epidermidis* 1457 in BM medium containing various concentrations of glucose. SAE, WT and SAEC were grown with or without 0.125%, 0.25%, 0.5%, and 1% (g/mL) glucose for 24 h. Then, the cultures were centrifuged. The pH of the supernatant was determined by using a pH meter (FE20, Mettler-Toledo, Shanghai, China).

**Figure 7:**
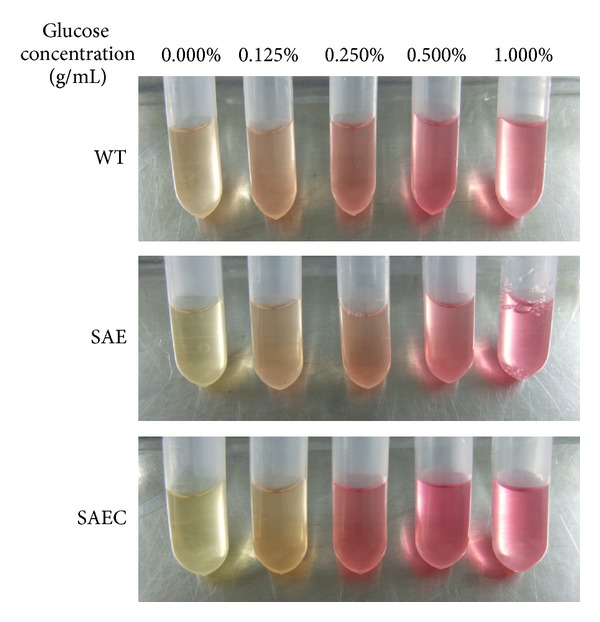
Methyl red test. SAE, WT, and SAEC were grown with or without 0.125%, 0.25%, 0.5%, and 1% (g/mL) glucose for 24 h. Five drops of methyl red were added to the culture supernatant and then gently rolled to disperse the methyl red. The supernatant will turn yellow at neutral pH but turn red at pH < 4.0.

Glucose is oxidized to pyruvate (in aerobic condition) or lactate (in anaerobic condition) in *S. epidermidis*, the pH value and the pH indicator methyl red may reflect the utilization efficiency of glucose. After 24 h, 0.25% glucose decreased the pH value of WT growth medium to 7.0 (lower than 7.4) and maintained the pH value of SAE and SAEC to 8.07 and 7.5 (higher than 7.4), respectively (Figure 6). For the methyl red test, SAE showed the lowest degree of red when cultured in BM medium containing 0.25% glucose, compared to WT and SAEC, indicating that SAE possesses the low utilization efficiency of glucose (Figure 7).

## 4. Discussion

Staphylococci biofilm formation and autolysis are influenced by a variety of different factors such as glucose, NaCl, pH, temperature, and growth phase, suggesting the existence of a mechanism that can sense environmental conditions [18]. In *S. epidermidis*, the two-component signal transduction system (TCS) SaeRS has been proven to negatively regulate biofilm formation and autolysis [12].

To further illustrate the regulation mechanism of SaeRS on biofilm formation and autolysis, two-dimensional gel electrophoresis (2-DE) was performed to analyze the expression of *S. epidermidis *proteins regulated by TCS SaeRS. In preliminarily work, the appropriate pH range for the first dimension isoelectric focusing (IEF) was determined using a 24 cm IPG strip of pH 3–10. Since most of the protein spots were present in the pH range of 4–7 (data not shown), subsequent 2-DE utilised 24 cm IPG strips of pH 4–7 for better separation and resolution. The amounts of protein and staining procedure used in the experiment were also empirically optimised. It was found that 120 *μ*g protein samples were required for the optimal silver staining.

Protein profile analysis of the *saeRS* mutant and the wild-type strain found 55 differentially expressed proteins in the present study. The transcription of four genes taking part in glycolysis was downregulated—*gapA1*, *eno*, *fbaA*, and *pyk*—indicating decreased glycolytic activity of SAE. We also found that the expression of catabolite control protein A (CcpA) was down-regulated in SAE. CcpA inhibits the genes expression involved in tricarboxylic acid cycle (TCA), such as *acnA* gene (encoding Aconitase) in *S. epidermidis *[19]. The formation of *S. epidermidis* biofilms is enhanced by conditions that repress tricarboxylic acid (TCA) cycle activity [19], indicating that there are other mechanisms involved in SaeRS-mediated biofilm formation.

*S. epidermidis saeRS* deletion mutant showed increased biofilm-forming ability in our previous study, and the mechanism includes increased extracellular DNA which may be caused by enhanced autolytic activity [12]. In the present study, appropriate concentration of glucose promotes the biofilm-forming ability of *S. epidermidis*, while excess glucose inhibits its biofilm-forming ability. In *S. epidermidis*, the effect of excess glucose on the metabolome was essentially identical to the effect of TCA cycle inactivation [20]. The formation of *S. epidermidis *biofilms may be enhanced by conditions that repress tricarboxylic acid (TCA) cycle activity. SAE showed strongest survival ability in BM medium containing 0.25% (g/mL) glucose; this may be caused by the low acidity of the growth medium. SAE showed lower pH value than WT grown in medium without glucose and higher pH value in medium with glucose, indicating the low ability of utilization of glucose in SAE.

PIA synthesis is important for biofilm formation [21–23]. In our previous study, no obvious difference in PIA production was observed between SAE and WT. PIA may not be the main cause of enhanced biofilm forming ability of SAE. The enhanced expression of accumulation-associated protein (identified in the 2-DE experiment) may play important role in SAE biofilm formation. In the present study, we have shown that glucose significantly promotes the biofilm formation of SAE, while having little effect on that of WT. The higher pH value that resulted from the low glucose utilization efficiency of SAE may also contribute to the biofilm formation. In addition, we have detected that high viability of SAE incubated in glucose excess medium, which may be caused by the high pH value, may also contribute to the biofilm formation.

## 5. Conclusions

TCS SaeRS positively regulates glucose metabolism in *S. epidermidis*. The repression of glucose utilization in SAE leads to decreased acidity of growth medium which may contribue to the higher viability and the enhanced biofilm forming ability. The present study suggests that in* S. epidermidis*, the external factor glucose plays an important role in the biofilm formation and viability regulated by SaeRS. The study will be helpful for treatment of *S. epidermidis* infection from the viewpoint of metabolic modulation dependent on two-component signal transduction system SaeRS.

## Figures and Tables

**Table 1:** Oligonucleotide primers used for RT real-time PCR.

Target gene	Gene description	GenBank accession no.	Primer*	Primer sequence	Location	Function
*gyrB *	DNA gyrase, B subunit	57636585	gyrBF gyrBR	CTTATATGAGAATCCATCTGTAGG AGAACAATCTGCCAATTTACC	1110–1263	House keeping gene

*pta *	Phosphate acetyltransferase	57866177	*ptaF* *ptaR *	AGGTTCTGCTAAATCGGATGATG GGTGCTTTCTTCTCTGCTACG	767–922	Catalyzes the conversion of acetyl-CoA and phosphate to CoA and acetyl phosphate

*gapA-1 *	Glyceraldehyde 3-phosphate dehydrogenase	57866427	*gapA-1F* *gapA-1R *	ACACAAGACGCACCTCAC TCTGGAATAACTTTACCGATAGC	709–821	Catalyzes the formation of 1,3-bisphosphoglycerate from glyceraldehyde-3-P in glycolysis

*eno *	Enolase; phosphopyruvate hydratase	57866440	*enoF* *enoR *	GCCAATTATTACAGATGTTTACG CAGTAGAAGCACCAGAAGG	200–327	Catalyzes the formation of phosphoenolpyruvate from 2-phospho-D-glycerate in glycolysis

*sucD *	Succinyl-CoA synthetase alpha subunit	57866714	*sucDF* *sucDR *	AGAAGAAGAAGCGGCACAATG GCAGTTTCAACACCACAATCG	779–958	Catalyzes the only substrate-level phosphorylation in the TCA cycle

*icd *	Isocitrate dehydrogenase	57867183	*icdF* *icdR *	ACTATATCTCTGATGCTCTTG TTCTGATGACGGATTAACC	1118–1272	Converts isocitrate to alpha ketoglutarate

*pyk *	Pyruvate kinase	57867187	*pykF* *pykR *	CGAGTTTCAGTGTTAGGTC CGTAGAGGTTAATTGATTATTCC	4–159	Catalyzes the formation of pyruvate from phosphoenolpyruvate in glycolysis

*fbaA *	Fructose-bisphosphate aldolase	57867608	*fbaAF* *fbaAR *	TGCTGATGGCGTTATCTATG CCGTGTAATACTAAAGGTAATCC	575–757	Catalyzes the formation of glycerone phosphate and glyceraldehyde 3-phosphate from fructose 1,6-bisphosphate

*F: forward primer; R: reverse prime.

**Table 2:** Protein expression regulated by *saeRS* in *S. epidermidis*.

Spot number^a^	Predicted MW (Da)	Predicted pI	Protein hit	Mowse Score^b^/number of match peptides	Accession number^c^	Fold change (2D: WT/SAE)
Upregulated protein spots						
179	18124	4.6	PTS system glucose-specific enzyme II A component [*Staphylococcus epidermidis* ATCC 12228]	215/13	27468033	1/4
175	18124	4.6	PTS system glucose-specific enzyme II A component [*Staphylococcus epidermidis* ATCC 12228]	156/11	27468033	1/4
2	131887	4.73	Accumulation-associated protein [*Staphylococcus epidermidis* RP62A]	88/6	7242804	1/10
121	131887	4.73	Accumulation-associated protein [*Staphylococcus epidermidis* RP62A]	190/16	7242804	1/10
17	131887	4.73	Accumulation-associated protein [*Staphylococcus epidermidis* RP62A]	72/6	7242804	1/10
19	131887	4.73	Accumulation-associated protein [*Staphylococcus epidermidis* RP62A]	124/9	7242804	1/10
30	131887	4.73	Accumulation-associated protein [*Staphylococcus epidermidis* RP62A]	187/11	7242804	1/10
33	131887	4.73	Accumulation-associated protein [*Staphylococcus epidermidis* RP62A]	127/7	7242804	1/10
1	131887	4.73	Accumulation-associated protein [*Staphylococcus epidermidis* RP62A]	83/9	7242804	1/10
74	35066	4.72	Phosphate acetyltransferase [*Staphylococcus epidermidis* ATCC 12228]	360/19	27467277	1/5
177	33131	5.18	Cysteine synthase [*Staphylococcus epidermidis* ATCC 12228]	110/15	27469188	1/2
93	33200	4.7	5-Oxo-1,2,5-tricarboxylic-3-penten acid decarboxylase [*Staphylococcus epidermidis* ATCC 12228]	83/9	27467583	1/10
222	19971	4.4	Heat shock protein 60 [*Staphylococcus muscae*]	81/6	17224365	1/1.5
326	15342	4.74	Hypothetical protein SE0591 [*Staphylococcus epidermidis* ATCC 12228]	174/10	27467509	1/2
265	18685	4.5	Hypothetical protein SE1560 [*Staphylococcus epidermidis* ATCC 12228]	89/5	27468478	1.5/1

Downregulated protein spots						
96	31679	5.35	Succinyl-CoA synthetase alpha subunit [*Staphylococcus epidermidis* ATCC 12228]	113/9	27467842	A/N^d^
412	41998	4.83	Succinyl-CoA synthetase beta subunit [*Staphylococcus epidermidis* ATCC 12228]	80/11	27467841	A/N
272	41998	4.83	Succinyl-CoA synthetase beta subunit [*Staphylococcus epidermidis* ATCC 12228]	74/14	27467841	A/N
143	31679	5.35	Succinyl-CoA synthetase alpha subunit [*Staphylococcus epidermidis* ATCC 12228]	99/10	27467842	7/1
307	41998	4.83	Succinyl-CoA synthetase beta subunit [*Staphylococcus epidermidis* ATCC 12228]	134/19	27467841	A/N
293	66196	4.99	D-Fructose-6-phosphate amidotransferase [*Staphylococcus epidermidis* ATCC 12228]	130/16	27468669	A/N
290	34981	4.66	Oxidoreductase ion channel [*Staphylococcus epidermidis* ATCC 12228]	74/9	27467285	6/1
504	32490	5.17	Elongation factor Ts [*Staphylococcus epidermidis* ATCC 12228]	159/17	27467851	A/N
234	18748	6.04	Putative vls recombination cassette Vls15 [*Borrelia burgdorferi*]	77/7	2039294	A/N
264	36442	5.29	Catabolite control protein A [*Staphylococcus epidermidis* RP62A]	75/9	57867258	A/N
84	52589	5.63	Inositol-monophosphate dehydrogenase [*Staphylococcus epidermidis* ATCC 12228]	92/16	27469266	A/N
260	22329	4.59	Hexulose-6-phosphate synthase, putative [*Staphylococcus epidermidis* RP62A]	124/11	57866142	A/N
386	34713	5.04	Glycerate dehydrogenase [*Staphylococcus epidermidis* RP62A]	101/13	57867831	A/N
754	47217	4.58	Enolase [*Staphylococcus epidermidis* ATCC 12228]	84/14	27467479	6/1
194	34185	4.61	Putative manganese-dependent inorganic pyrophosphatase [*Staphylococcus epidermidis* RP62A]	190/19	57867405	A/N
163	33131	5.18	Cysteine synthase [*Staphylococcus epidermidis* ATCC 12228]	88/13	27469188	1.5/1
424	66196	4.99	D-Fructose-6-phosphate Amidotransferase [*Staphylococcus epidermidis* ATCC 12228]	77/10	27468669	A/N
171	26556	4.51	30S ribosomal protein S1 [*Staphylococcus epidermidis* ATCC 12228]	137/12	27468083	10/1
162	42075	4.62	DNA polymerase III Subunit beta [*Staphylococcus epidermidis* ATCC 12228]	79/11	27466920	1.5/1
94	62994	5.24	Pyruvate kinase [*Staphylococcus epidermidis* ATCC 12228]	230/20	27468291	A/N
415	46590	5.01	Isocitrate dehydrogenase [*Staphylococcus epidermidis* ATCC 12228]	72/7	27468288	7/1
82	66105	4.57	DnaK protein [*Staphylococcus epidermidis* RP62A]	257/17	57867037	5/1
96	66105	4.57	DnaK protein [*Staphylococcus epidermidis* RP62A]	77/7	57867037	5/1
72	66105	4.57	DnaK protein [*Staphylococcus epidermidis* RP62A]	84/10	57867037	5/1
21	66105	4.57	DnaK protein [*Staphylococcus epidermidis* RP62A]	157/12	57867037	5/1
74	66105	4.57	DnaK protein [*Staphylococcus epidermidis* RP62A]	88/14	57867037	A/N
142	33031	4.82	Fructose-bisphosphate aldolase [*Staphylococcus epidermidis* ATCC 12228]	261/24	27469074	3/1
481	14906	5.96	Urease beta subunit [*Staphylococcus epidermidis* ATCC 12228]	218/18	27468780	4/1
90	36168	4.83	Glyceraldehyde-3-phosphate dehydrogenase [*Staphylococcus epidermidis* ATCC 12228]	123/13	27467475	A/N
83	52589	5.63	Inositol-monophosphate dehydrogenase [*Staphylococcus epidermidis* ATCC 12228]	86/13	27469266	A/N
45	52589	5.63	Inositol-monophosphate dehydrogenase [*Staphylococcus epidermidis* ATCC 12228]	106/16	27469266	A/N
44	52589	5.63	Inositol-monophosphate dehydrogenase [*Staphylococcus epidermidis* ATCC 12228]	205/27	27469266	A/N
101	48702	4.28	Trigger factor [*Staphylococcus epidermidis* ATCC 12228]	89/13	27468268	3/1
148	51524	4.62	ATP synthase subunit B [*Staphylococcus epidermidis* ATCC 12228]	96/6	27468618	4/1
148	51524	4.62	ATP synthase subunit B [*Staphylococcus epidermidis* ATCC 12228]	107/7	27468618	4/1
227	20203	5.71	Immunodominant antigen B [*Staphylococcus epidermidis* ATCC 12228]	81/8	27468493	1.5/1
237	35151	5.42	Hypothetical protein SE1260 [*Staphylococcus epidermidis* ATCC 12228]	112/16	27468178	4/1
261	35151	5.42	Hypothetical protein SE1260 [*Staphylococcus epidermidis* ATCC 12228]	111/16	27468178	1.5/1
654	17753	5.55	Hypothetical protein SE2391 [*Staphylococcus epidermidis* ATCC 12228]	126/10	27469309	35/1
320	19182	4.69	Hypothetical protein SE0264 [*Staphylococcus epidermidis* ATCC 12228]	113/15	27467182	10/1

^a^Spots numbers correspond to the numbers in Figure 2.

^b^Protein score was from MALDI-TOF/TOF identification. The proteins that had a score great than 72 (*P* < 0.05) were considered statistically significant.

^c^Accession no. is the MASCOT result of MALDI-TOF/TOF searched from the NCBInr database.

^d^A/N indicates the spot detected only in the protein extracts of *S. epidermidis* 1457 wild-type strain.

## Abbreviations

WT: *S. epidermidis* wild-type
SAE: *S. epidermidis saeRS* mutant
SAEC: *S. epidermidis saeRS* complemental strain
2-DE: Two-dimensional gel electrophoresis
TCA: Tricarboxylic acid
PIA: Polysaccharide intercellular adhesion
TCS: Two-component signal transduction system
CFU: Colony-forming unit.
